# Editorial: Cellular and molecular characteristics of the pre-metastatic niche

**DOI:** 10.3389/fonc.2023.1298958

**Published:** 2023-10-24

**Authors:** Abhishek Tyagi

**Affiliations:** Department of Cancer Biology, Wake Forest University School of Medicine, Winston-Salem, NC, United States

**Keywords:** pre-metastatic niche, organotropism, immunosuppression, lymphangiogenesis and vascular permeability, inflammation and reprogramming

Tumor metastasis remains a prominent contributor to cancer-related mortality, underscoring the critical imperative of identifying pivotal molecular and cellular constituents involved at each stage of the metastatic cascade (1). Consequently, it is of paramount importance to devise novel strategies aimed at both the prevention and management of tumor metastasis. Indeed, primary tumors can proactively modulate the local microenvironment in distant organs to facilitate the colonization of tumor cells even before their arrival. This phenomenon aligns with the “seed and soil” hypothesis (2), which has significantly enriched our comprehension of tumor metastasis and explains the organ-specific patterns observed in metastatic spread. Furthermore, Fidler’s discoveries not only bolstered Paget’s theory but also rekindled interest in the fundamental question that initially captivated Paget (3, 4): Why tumor cells preferentially emerge as disseminated tumor cells (DTCs) within specific organs is a multifaceted and still inadequately understood question, and grasping the underlying mechanisms is of paramount importance in cancer research. This intriguing phenomenon of organ specificity in metastasis, termed organotropism, represents one of the most compelling and unresolved puzzles in the field of cancer research.

A growing body of evidence now substantiates the notion that the primary tumor can enhance the process of metastasis by instigating the development of a conducive microenvironment in a secondary organ site. This specialized environment is referred to as the “pre-metastatic niche (PMN)” (5–7). The formation of the PMN is a stepwise process driven by a combination of systemic factors and vesicles secreted by primary tumor-derived components, tumor-mobilized bone-marrow-derived cells (BMDCs), and the local stromal microenvironment of the host (or future metastatic organ components). They work jointly with cellular components to initiate, polarize, and establish PMN in future metastatic organs (8–10). PMN formation commences with localized changes, including the induction of vascular permeability, remodeling of the stroma, and alterations in the extracellular matrix followed by systemic effects on the immune system (11). Many molecular and cellular components contributing to PMN formation have been identified in different tumor models (12–14).

A variety of cellular and molecular components have been confirmed to play essential roles in the formation of the PMN, endowing the niche with six distinct characteristics that promote metastasis. However, a multitude of unresolved questions surround the intricacies of PMN formation, its functional dynamics, and its overall significance. Additionally, the task of translating these foundational research findings into effective clinical practices presents a multifaceted challenge (15).

A multitude of critical inquiries surrounding PMN formation and its implications for tumor metastasis demand thorough investigation. Firstly, understanding the precise spatiotemporal regulatory functions of PMN characteristics at different stages of tumor metastasis is paramount. Secondly, the conditions under which primary tumors create PMN, whether universally or selectively in contexts like inflammation or hypoxia, require better understanding. Thirdly, the contributions of host systemic and local immunosuppression and inflammation to PMN formation and its metastasis-promoting function are essential to discern. Fourthly, establishing the causal relationship between the primary tumor microenvironment and PMN formation during metastasis initiation is crucial. Moreover, monitoring the progression of PMN formation processes may offer opportunities to identify novel and robust biomarkers, enabling the early detection of metastatic development before the emergence of overt metastases. This proactive approach to cancer monitoring and diagnosis could be instrumental in improving patient outcomes and guiding more targeted therapeutic interventions.

Gaining a deeper understanding of the mechanisms underpinning PMN formation and characterizing their impact on tumor metastasis holds immense promise for unveiling novel therapeutic strategies in the battle against metastatic cancers. The development of precise technologies and innovative approaches aimed at detecting PMNs within distant organ sites in patients has the potential to bring about a transformative shift in cancer treatment. This could pave the way for proactive interventions designed to impede the progression of metastasis, offering patients a more favorable prognosis. The concept of the PMN represents a groundbreaking paradigm shift in our comprehension of metastasis initiation, and our capacity to combat metastatic disease can undergo substantial enhancement through a profound understanding of the pathological processes occurring before the development of macroscopic metastases. Finally, effectively targeting the PMN to prevent metastasis and assessing the impact of current therapies like radiation, chemotherapy, targeted therapy, and immunotherapy on the niche represent pivotal avenues for future research with the potential to improve cancer patient outcomes significantly (16).

The articles featured in this Research Topic will serve as a foundation for gaining valuable mechanistic insights and perspectives into the intricate process of metastasis. One of the articles explores the enigmatic interplay between cancer and the internal physiologic milieu, potentially reshaping paradigms pertinent to cancer prevention and therapeutic modalities [Paul and Nedelcu et al.]. Another article highlights the significance of spontaneous osteoclastogenesis as a risk factor for bone metastasis, especially in advanced luminal A-type breast cancer patients, offering new perspectives on disease progression [Fernandez Vallone et al.]. Additionally, a study reveals LOXL1 and LOXL4 as emerging target genes regulated by the Zn^2+^-bound form of ZEB1 in triple-negative breast cancer cells, contributing to their invasive capabilities, a hallmark feature for establishment of PMN in distal organs [Hirabayashi et al.]. In parallel, another study delves into the distinctive role of lysyl oxidase-like 4 (LOXL4) in breast cancer progression, that occurs via an interaction with annexin A2 and integrin β-1 on the cell surface [Komalasari et al.]. Finally, an article explores the pivotal role of the extracellular matrix in the orchestration of PMN, enriching our comprehension of the mechanisms underlying cancer metastasis [Patras et al.].

The compilation of articles offers a glimpse of future directions in developing a framework for potential clinical and translational strategies within the domain of cancer metastasis therapy research. We hope that these articles will captivate and inform the readership of Frontier—an audience deeply dedicated to eradicating a threat that is, indeed, awaiting its abolition.

## Author contributions

AT: Conceptualization, Writing – original draft, Writing – review & editing.
